# Supplementary material for: The interactome of intact mitochondria by cross-linking mass spectrometry provides evidence for coexisting respiratory supercomplexes
Source: Mol Cell Proteomics. 2017 Dec 8;17(2):216–32. doi: 10.1074/mcp.RA117.000470 (PMC5795388; doi:10.1074/mcp.RA117.000470)
Supplement: Supplemental Data [file supp_RA117.000470_133922_0_supp_23978_fzffwf.zip › spectra_annotation/mito_DR_spectra_annotation/456-1-1-1-24-1.pdf]

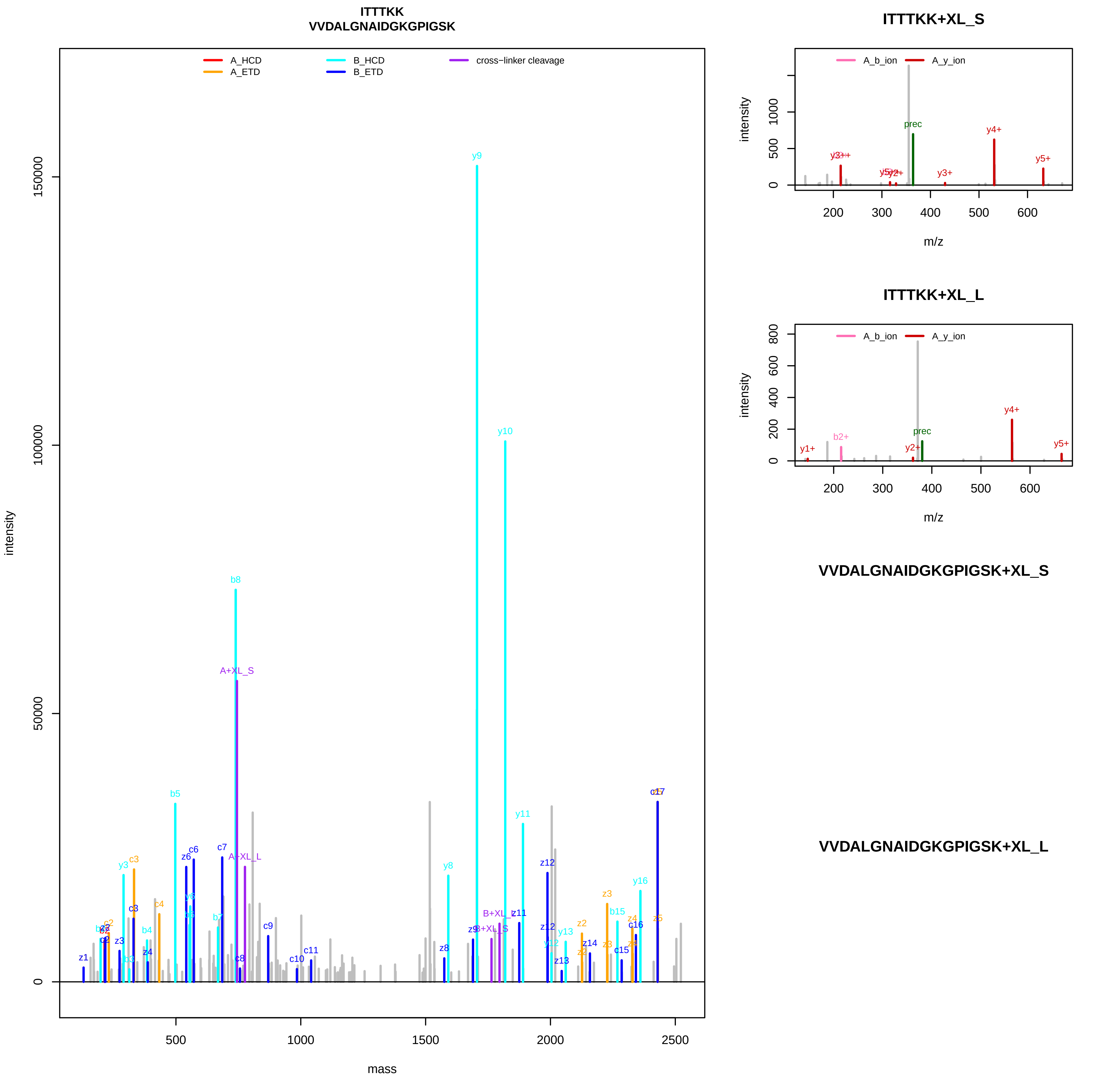
ITTTKK
VVDALGNAIDGKGPIGSK
A_HCD
A_ETD
B_HCD
B_ETD
cross−linker cleavage
intensity
mass
y9
y10
b8
A+XL_S
A+XL_L
B+XL_L
B+XL_S
b5
y11
y8
y16
c17
z12
c3
y3
c6
z6
c7
z11
z8
c9
c11
c10
z13
z14
c15
c16
b15
y13
y12
z3
z4
z5
z2
b4
c4
b7
c8
z1
c2
ITTTKK+XL_S
ITTTKK+XL_L
A_b_ion
A_y_ion
prec
y4+
y5+
y3+
y2+
y1+
b2+
m/z
VVDALGNAIDGKGPIGSK+XL_S
VVDALGNAIDGKGPIGSK+XL_L
